# ‘Broken hospital windows’: debating the theory of spreading disorder and its application to healthcare organizations

**DOI:** 10.1186/s12913-018-3012-2

**Published:** 2018-03-22

**Authors:** Kate Churruca, Louise A. Ellis, Jeffrey Braithwaite

**Affiliations:** Centre for Healthcare Resilience and Implementation Science, Australian Institute of Health Innovation, Macquarie University, Sydney, NSW 2109 Australia

**Keywords:** Broken windows theory, Organizational theory, Quality of care, Patient safety, Harm, Violations, Healthcare, Hospitals

## Abstract

**Background:**

Research in criminology and social-psychology supports the idea that visible signs of disorder, both physical and social, may perpetuate further disorder, leading to neighborhood incivilities, petty violations, and potentially criminal behavior. This theory of ‘broken windows’ has now also been applied to more enclosed environments, such as organizations.

**Main text:**

This paper debates whether the premise of broken windows theory, and the concept of ‘disorder’, might also have utility in the context of health services. There is already a body of work on system migration, which suggests a role for violations and workarounds in normalizing unwarranted deviations from safe practices in healthcare organizations. Studies of visible disorder may be needed in healthcare, where the risks of norm violations and disorderly environments, and potential for harm to patients, are considerable. Everyday adjustments and flexibility is mostly beneficial, but in this paper, we ask: how might deviations from the norm escalate from necessary workarounds to risky violations in care settings? Does physical or social disorder in healthcare contexts perpetuate further disorder, leading to downstream effects, including increased risk of harm to patients?

**Conclusions:**

We advance a model of broken windows in healthcare, and a proposal to study this phenomenon.

## Background

Despite the increasing focus on quality and safety in healthcare over the last two decades, rates of unwarranted variations, adverse events and preventable harm to patients remain high [1, 2]. One of the factors contributing to problems in the quality and safety of patient care is the way in which ‘substandard care’ and risky behavior among healthcare professionals are often ‘easily normalized within the organizational routines and culture of care services, especially if these services [are] under-resourced’ ([3], p. 201, [4]). In this paper, we introduce a novel approach to conceptualizing these normalization processes within healthcare, one which may shed new light on the relationships between the physical and social environment of hospitals, on the one hand, and the psychology and behaviors of the health professionals who work within them, on the other. Adapted from sociology and social psychology, this approach focuses on the way disorder, operationalized as both social and physical, may perpetuate itself, and spread into individual behaviors. It is most commonly called broken windows theory (BWT).

## Main text

### Broken windows: A theory of spreading disorder in neighborhoods

Almost 40 years ago, Wilson and Kelling famously used broken windows as a metaphor for disorder within neighborhoods, arguing that ‘if a window in a building is broken and is left unrepaired, all of the rest of the windows will soon be broken’ [5]. Hence, BWT proposes that visible signs of neighborhood disorder (e.g., broken windows, graffiti, litter, or boarded-up buildings) lead to further disorderly behavior (e.g., neglect of surrounds, vandalism, or antisocial activities), because they provide cues to the kinds of actions that are routinely tolerated, and which inhabitants might themselves mimic, or get away with. Signs of disorder are also thought to convey to residents of a neighborhood potential problems of safety in the area, leading to their withdrawal from public spaces, and thereby a reduction in informal social control, which can further perpetuate this effect [5]. BWT, as a social-psychological theory of urban decline, has informed fields such as criminology, sociology, and public health [6], as well as being used, highly controversially, as a rationale for practices of zero-tolerance policing [7].

Conceptual and methodological issues have been advanced in the study of broken windows, with Sampson and Raudenbush [8] noting that much of this research has assumed disorder to be an essential and objective phenomenon, while they argue that context and cultural stereotypes (i.e., racism, classism) have as much to do with what is constructed as ‘disorder’, and why it is construed as a problem. Other points of contention concern whether it is better to study residents’ perceptions or to systematically observe disorder, and whether and in what ways physical disorder is conceptually distinct from social disorder [9].

**Fig. 1:**
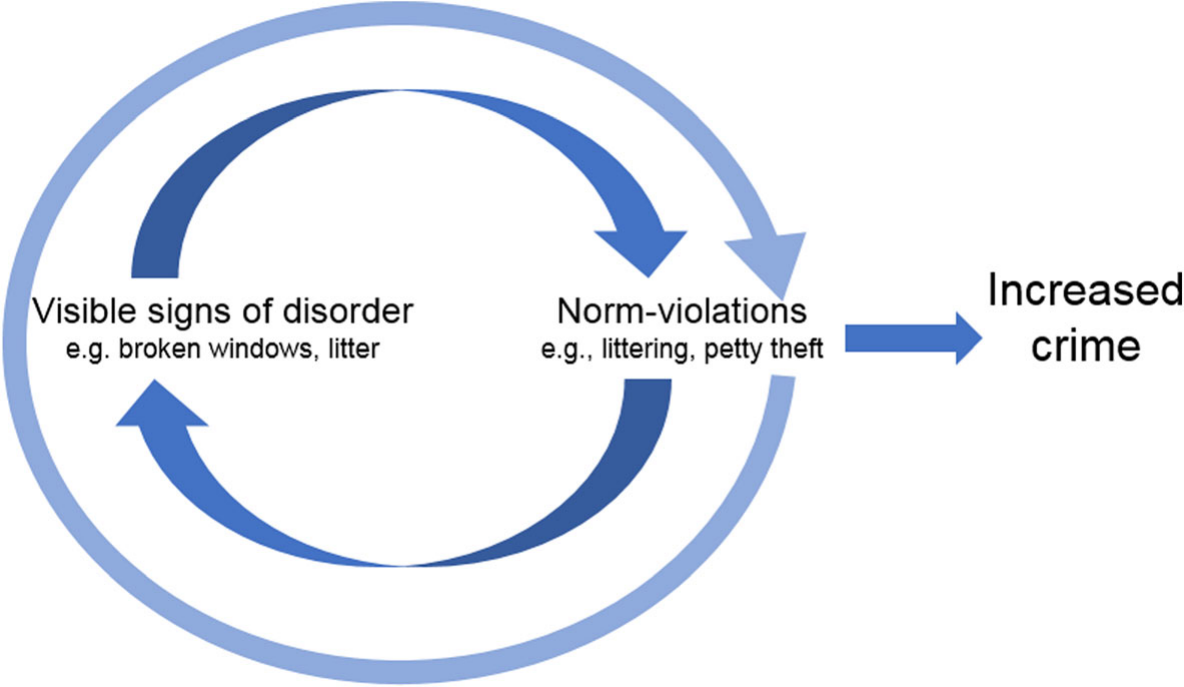
As suggested by BWT, visible signs of disorder, such as broken windows or litter, signal to inhabitants of a neighborhood a lack of social control. This leads them to violate social norms, rules and laws (e.g., littering, vandalism); these behaviors then perpetuate further visible signs of disorder and other norm-violations. Over time, the reinforcing relationship of disorder and violations is thought to result in increased crime

These issues notwithstanding, one potentially fruitful way of accounting for a BWT-type effect has been to understand disorder as perpetuating ‘norm violations’ [10–12]. Violations are when people deliberately break the rules or fail to comply with procedures and are often driven by a psychological ‘balance sheet’ of perceived costs versus benefits of the violation [13]. The norm-violation approach to BWT suggests that disorder spreads because in situations where the potential cost of breaking a social norm is low (despite that there may be benefits to this deviance), individuals rely more on contextual cues. They use these cues to assess the likelihood and severity of them receiving sanction for performing the same or similarly inappropriate behaviors as those that are already evident in the environment. Hence, as shown in Fig. 1, visible disorder (e.g., litter) not only leads to increased likelihood of violating the same norm (i.e., more littering), but can escalate, having ‘spillover effects where violations of one norm foster non-compliance’ to other norms, such as jaywalking or vandalism ([12], p. 101).

According to this approach, disorder is evidence of a lack of social control, while the presence of shared expectations, values and trust among inhabitants of a space (i.e., often termed as ‘local social capital’ or ‘collective efficacy’), is thought to encourage attempts to maintain compliance with norms and the valuing of personal and communal reputation, such as by sanctioning others who violate the rules [12, 14]. Therefore, a neighborhood high in social capital is expected to exhibit stronger social control and less propensity for ‘broken windows’ in the first instance. However, this has been suggested to lead to an interaction effect—between disorder and social capital—with areas of higher-levels of social capital manifesting a stronger broken windows effect due to the greater salience of contextual cues in an otherwise well-controlled environment and the tendency for social cohesion (i.e., conformity to others behavior) among those with high social capital [12].

### Beyond the neighborhood: Hospital ‘broken windows’

Recently, BWT has been applied beyond neighborhoods to smaller, more enclosed and non-anonymous environments, such as workplaces. Ramos and Torgler [15], for example, found that academics littered more frequently when their kitchen common room was physically disordered and that this occurred even with an observer present (i.e., higher risk of being sanctioned). Thus, the presence of disorder (e.g., cutlery and crockery in the sink, discarded packaging left on benches) increased the probability of staff violating a target norm (i.e., littering). This finding suggests the potential utility in extending BWT beyond just the neighborhood context and that it might explain some of the low-consequence but inappropriate behaviors we observe in workplaces (e.g., the misappropriation of office teaspoons; [16]).

However, in other types of workplaces, such as hospitals and similar sites of healthcare delivery, even apparently small deviations or violations (i.e., taking short cuts in reading a drug dose, or in washing hands) in workplace norms may have life or death consequences [4]. At the same time, the limited evidence available suggests that such violations are remarkably common, often tolerated among healthcare workers, and tend to be performed because they provide some other benefit (e.g., efficiency, increased patient-centeredness, personal enhancement) despite being a deviation from the norm [4, 17, 18]. This begs the questions, is a broken-windows type effect something we might observe in a hospital environment? And if so, might such an effect have downstream implications on the delivery of care to patients?

In the past, there have been brief mentions of a broken-windows type effect in hospitals. For example, in questioning ‘Why is patient safety so hard?’, Dixon-Woods argues that similar to how petty infractions are thought to encourage criminal behavior in BWT, ‘trivial distractions are consequential for the overall climate of safety’ in healthcare [19]. Empirical research in healthcare has also drawn upon BWT; however, this has been limited to how BWT might explain the perpetuation of violence among healthcare professionals and patients, therefore taking a rather narrow view of social disorder, and with no consideration of physical disorder, or of the effects on patient outcomes [20]. However, from a review of the literature, there appears to be a number of reasons to support the full application of BWT to the hospital context. Acute healthcare delivery is a high-hazard industry; however, unlike many other such types of industries (nuclear power, aviation), there are few enforced rules; instead, guidelines, protocols and policies are applied flexibly, with staff often working independently and able to exercise clinical judgement [21–23]. In such an environment, certain behaviors among staff are less likely to encounter formal or informal control; however, such ‘workarounds’ may also conflict with the organizationally prescribed or intended procedures (i.e., the norm; [17, 24]).

Take the case of noncompliance to hand hygiene procedures. Poor hand hygiene is one of the most controllable causes of hospital-acquired infection [25]. Erasmus, Brouwer, van Beeck, et al. [26] investigated why there is often a lack of compliance among hospital workers. They found that despite the positives, there were also some disadvantages to hand-washing, such as leading to dry, sore hands, and requiring considerable time. Furthermore, there was a lack of social control for this practice, with staff feeling uncomfortable with broaching ‘norm-violations’ with colleagues. Indeed, junior members of staff often followed the behaviors of those in senior positions. In a similar vein, de Saint Maurice, Auroy, Vincent and Amalberti [27] showed that compliance with a safety policy in anesthesia eroded over a year, seemingly beginning with violations by more senior members of staff.

Amalberti, Vincent, Auroy and de Saint Maurice [4] have proposed a framework for understanding the occurrence of violations in healthcare, which shares some similarities with BWT, involving, as it does, the ‘migration’ of the environment to one of ‘normal illegal’. That is, deviance among staff is increasingly tolerated, normalized and even required in the pressurized environment of patient care delivery, where trade-offs (e.g., safe behavior versus efficient behavior) are often part-and-parcel of the work getting done. This ‘system migration’ is also problematic; the tolerance of increasingly extreme norm-violations in this context may have effects on par with neighborhood criminality, by having the potential to cause patient harm or even death [28].

**Fig. 2:**
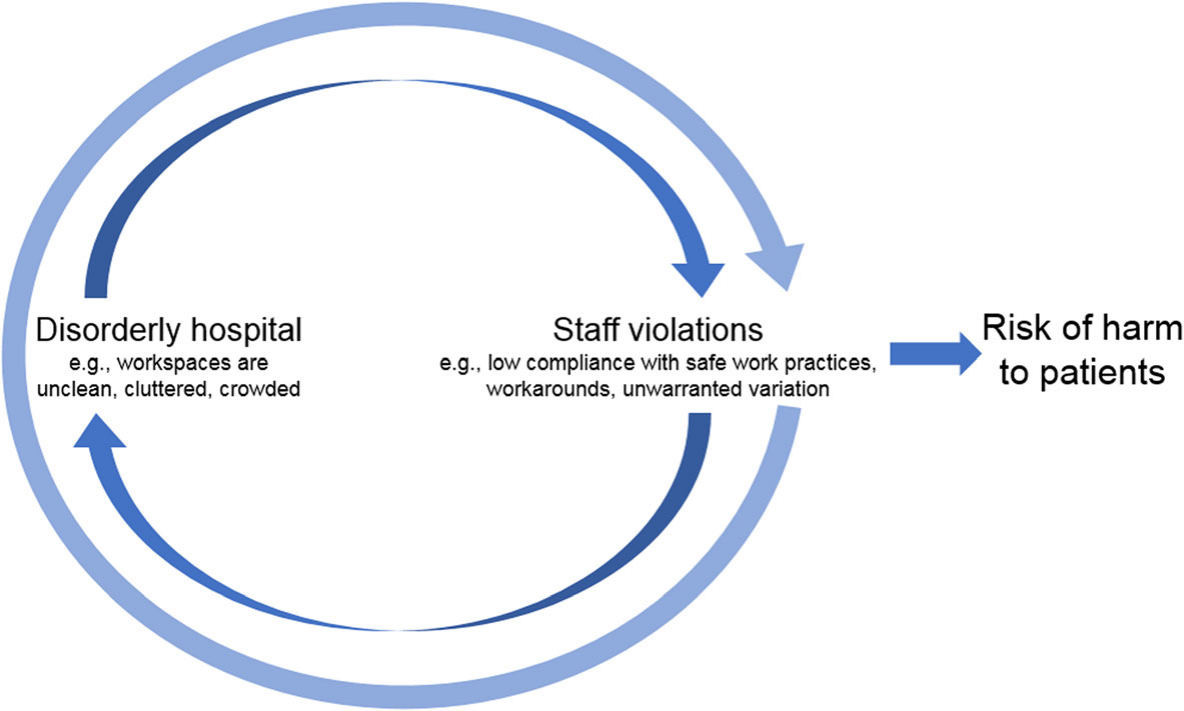
Adaptation of the premises of BWT to hospital context. Here visible signs of disorder in a healthcare facility signal a lack of control or concern for the environment. This may lead staff to violate minor rules of the workplace, such as hand hygiene rules or not securing medical equipment. These behaviors then perpetuate further visible signs of disorder and other staff violations. Over time, the reinforcing relationship between disorder and violations increases the risk of harm to patients as the system migrates to one of “normal illegal” [4]

Traditionally, BWT has also provided an explanation for the relationship between behavior and the physical environment (i.e., physical disorder). There is less evidence to support the idea that physical disorder perpetuates social disorder in a hospital environment, or has potential downstream effects such as on patient outcomes. However, the work environment is a recognized system through which safe and reliable care is provided to patients [29–31], with factors like architecture, noise and lighting known to affect the safety of care delivery [32]. Beyond issues of cleanliness, and elements of maintenance and design (e.g., number of hand washing basins, easy-to-clean surfaces) which we might expect to have a direct association with patient safety outcomes [33], there is also evidence for a relationship between the orderliness of workspaces and the way staff behave within them. Gershon, Karkashian, Grosch, et al. [34], in a survey of hospital safety climate, found that staffs’ perception of their workspaces as clean and orderly was strongly associated with their tendency to comply with safe work practices. This suggests the potential role of physical disorder in staffs’ compliance with safe work practices (i.e., norm-violations), which could detrimentally affect care delivery. Hence, we propose to adapt to a hospital context the theory of spreading disorder (Fig. 2).

If a broken windows effect could explain some of these documented issues in the delivery of safe care to patients, the potential implications of applying BWT to healthcare would be significant. It would point toward some very clear strategies for quality improvement, as well as ways to enhance patient and staff satisfaction, in ‘taking care of the little things’, for example, by keeping the physical environment clean and tidy. This is not to suggest a similarly ‘zero-tolerance’ approach to violations in healthcare as has been applied for neighborhood disorder, because often the very nature of the work—complex, time-pressured and dynamic—requires staff to make various forms of trade-offs, or engage in necessary workarounds [17, 35]. Rather, we must help ensure system migration does not go too far, that seriously unsafe work practices are not normalized, and that efforts are made on the part of key stakeholders (policymakers, managers, clinicians, patient bodies) to understand and address the reasons that critical violations or unwarranted variations in care occur [21, 22]. We are talking about achieving balance in a complex adaptive system, rather than imposing more controls or burdensome regulations on stretched staff [36, 37].

So, there is work to be done in studying whether BWT holds up in healthcare contexts: whether social and physical disorder perpetuate further disorder, and whether, and in what ways, they might affect outcomes. This is not a straightforward task. Just as studying BWT in neighborhood contexts has been a tricky endeavour, applying it to the hospital environment would pose both similar and unique challenges. For example, conceptually defining and then operationalising disorder, and making the distinction between social and physical disorder, remain challenges for both environments [9]. What counts as disorder in a hospital might be more a matter of general agreement, rather than some essential quality of the physical environment, just as the nature of error in healthcare is fluid, something open to contestation and negotiation [38]. Furthermore, the relationship of social control and social capital to disorder suggests that some of the constructs we are already interested in studying in healthcare settings, particularly clinical/medical engagement [39] and teamwork [40], might affect any relationship between norm-violations and disorder in hospitals. Engagement, for example, might lead staff to greater observance of norms even in the face of disorder, while teamwork could have the opposite effect, with group cohesion providing the conditions suitable for spreading disorder [12]. The point is we do not know whether these speculations hold, but it is an important enough issue for us to develop a research program to assess it.

Methodologically, the push toward naturalistic experimentally designed studies of BWT (e.g., [11, 12]) is not so easily accomplished in places like hospitals, where fiddling with disorder and watching whether that leads to staff breaking rules or deviating from guidelines involves far more potent ethical issues with potential deleterious effects on patient care. Hence, the first step in studying this phenomenon might, by necessity, be to look at the associations between orderliness and patient outcomes, and would initially involve addressing the challenge of arriving at some sort of rigorous way of measuring disorder specifically in the context of healthcare. Given the debate regarding subjective versus objective assessments of disorder, varying types of data collection (e.g., staff surveys and researcher-led structured observations) are likely targets. Into the future, we might consider how hospitals are situated within their neighborhoods and broader contexts (i.e., regional, national). Following Sampson and Raudenbush’s [8] contention that social stigma affects what is perceived as disorder, this could allow us to unpack more complex relationships among inequality, hospital disorder and patient and staff outcomes.

## Conclusions

As a novel way of studying hospitals and staff behavior, BWT broadly aligns with other trends in understanding healthcare quality and safety over the past two decades, including movement away from blaming individuals to a focus on systems [41], and recognition that context and culture matter [42, 43]. In its relatively intuitive categorization of the physical and social environment—through the concept of ‘disorder’—BWT might further move the needle in the direction of greater understanding of how healthcare works in practice.

There is much work ahead. However, the premise of BWT holds considerable value. The potential benefits are large, and the costs are minimal, so the question becomes: keeping things clean, tidy, orderly—where’s the harm?

## Abbreviation

BWT: Broken windows theory
